# Supplementary material for: Answering Clinical Questions Using Machine Learning: Should We Look at Diastolic Blood Pressure When Tailoring Blood Pressure Control?
Source: J Clin Med. 2022 Dec 15;11(24):7454. doi: 10.3390/jcm11247454 (PMC9785044; doi:10.3390/jcm11247454)
Supplement: Supplementary file 1 [file jcm-11-07454-s001.zip › jcm-2059975-SM.pdf]

Supplemental Material

Table S1. Baseline characteristics of the Systolic Blood Pressure Intervention Trial (SPRINT) participants, according to the treatment group.

	Intensive treatment	Standard treatment	P-value
N	4678	4683	
Age, years	67.9 (9.4)	67.9 (9.5)	0.9
Female, %	1684 (36)	1648 (35.2)	0.43
Black race, %	1454 (31.1)	1493 (31.9)	0.42
History of cardiovascular disease, %	940 (20.1)	937 (20)	0.9
History of chronic kidney disease, %	1330 (28.4)	1316 (28.1)	0.7
Body mass index, kg/m^2	29.9 (5.8)	29.8 (5.7)	0.40
Systolic blood pressure, mmHg	139.7 (15.8)	139.7 (15.4)	0.97
Diastolic blood pressure, mmHg	78.2 (11.9)	78 (12)	0.48
Current smoker, %	639 (13.7)	601 (12.8)	0.25
Estimated glomerular filtration rate, ml/min/1.73 m^2	71.8 (20.7)	71.7 (20.5)	0.9
Plasma glucose, fasting, mg/dl	99 (14)	99 (13)	0.9
Total cholesterol, mg/dl	190.2 (41.4)	190 (41)	0.9
High density lipoprotein cholesterol, mg/dl	52.9 (14.3)	55 (14.6)	0.7
Triglycerides, mg/dl	125 (86)	127 (95)	0.22
On aspirin, %	2406 (51.6)	2350 (50.4)	0.23
On statin, %	1978 (42.6)	2076 (44.7)	0.038

Variables are presented as mean values, standard deviation or number, followed by percentage.

Table S2. The Systolic Blood Pressure Intervention Trial (SPRINT) outcomes.

	Intensive treatment	Standard treatment	p-value
N	4678	4683	
Mean in-trial systolic blood pressure, mmHg	123.5 (8.6)	135.3 (7.2)	<0.001
Mean in-trial diastolic blood pressure, mmHg	69.1 (8.4)	75.1 (9.1)	<0.001
Original primary SPRINT composite endpoint, %	243 (5.2)	319 (6.8)	0.001
Redefined clinical composite endpoint*, %	204 (4.4)	257 (5.5)	0.012
Myocardial infarction, %	97 (2.1)	116 (2.5)	0.22
Acute coronary syndrome other than myocardial infarction, %	40 (0.9)	40 (0.9)	0.99
Stroke, %	62 (1.3)	70 (1.5)	0.5
Acute exacerbation of heart failure, %	62 (1.3)	100 (2.1)	0.003
Cardiovascular death, %	37 (0.8)	65 (1.4)	0.007
Overall death, %	155 (3.3)	210 (4.5)	0.004

Variables are presented as mean values, standard deviation or number, followed by percentage.

* In this report the composite clinical endpoint was redefined (by excluding acute exacerbation of heart failure, as compared to the original SPRINT report) as myocardial infarction, acute coronary syndrome other than myocardial infarction, stroke and cardiovascular death.
